# EXPLORING AGING DYNAMICS IN EGYPT: INSIGHTS FROM THE AL-SEHA PILOT STUDY

**DOI:** 10.1093/geroni/igae098.0152

**Published:** 2024-12-31

**Authors:** Sara Moustafa, Nada Gaballah, Reem Deif, Mohamed Salama

**Affiliations:** The American University in Cairo, Cairo, Al Qahirah, Egypt; The American University in Cairo, Cairo, Al Qahirah, Egypt; The American University in Cairo, Cairo, Al Qahirah, Egypt; The American University in Cairo, Cairo, Al Qahirah, Egypt

## Abstract

The AL-SEHA (A Longitudinal Study of Egyptian Healthy Aging) pilot study is an HRS-Around the World (ATW) longitudinal sister study in Egypt. This foundational phase aims to uncover critical insights into cognitive impairment, the prevalence of non-communicable diseases (NCDs), and the pivotal role of family support for older adults in Egypt. The cross-sectional pilot was conducted in five Egyptian governorates: Cairo, Dakahlia, Beni Suef, Suez, and Fayoum. Participant selection was based on convenient sample of Egypt’s population aged 50+. Data collection combined computer-aided personal interviewing (CAPI) with a Self-Completion Questionnaire (SCQ), and included comprehensive coverage of economic, health, and social domains including caregiving needs and arrangements. Initial findings highlight a significant association between cognitive impairment and NCDs, such as diabetes (62.3%), hypertension (75.8%), and musculoskeletal issues (61.3%). As in other populations, lower educational attainment had a significant negative association with cognitive impairment (p=0.0001), emphasizing socioeconomic factors’ impact on cognitive health. Strong family support networks were identified as key in mitigating cognitive decline, underscoring the cultural importance of family in the Egyptian context. The AL-SEHA pilot study provides a vital foundation for understanding the aging process in Egypt, highlighting the interplay between cognitive health, physical well-being, and socio-economic and cultural factors. Findings advocate for integrated healthcare strategies that address the multifaceted needs of the older population. Future research, building on this pilot phase, is essential to develop evidence-based policies and interventions to enhance the quality of life for Egypt’s aging population.

